# Ventricular assist device simulation to improve staff confidence and knowledge when caring for patients supported with the Berlin heart☆

**DOI:** 10.1051/ject/2025058

**Published:** 2026-06-19

**Authors:** Laura Valido, Vicky Duffy, Erica Rey, Matt Deitemyer, Tracy Heard, Jaqueline Pleiman, Richard Fernandez

**Affiliations:** 1 The Heart Center, Nationwide Children’s Hospital 700 Children’s Drive Columbus OH 43205 USA

**Keywords:** Ventricular Assist Device, Berlin Heart, Simulation, Cardiac Intensive Care

## Abstract

*Background*: Rapid growth of our mechanical cardiac support program, coupled with limited experience among bedside nursing staff in the care of patients supported with the Berlin Heart (BH VAD), created the need for a targeted educational intervention. We designed an educational module combining didactic sessions with high-fidelity simulation of a patient supported with a BH VAD. *Methods*: A novel, high-fidelity BH VAD simulator was designed and tested by our team. Members of the cardiothoracic intensive care unit (CICU) staff participated in didactic sessions and high-fidelity simulations. Pre- and post-experience surveys were administered to gauge staff confidence and basic knowledge regarding caring for a patient supported with a BH VAD. Follow-up surveys were administered 6–8 weeks after the educational module was completed. *Results*: 82 total staff members participated in the simulation. There were significant improvements in feelings of being prepared to care for a patient supported with a BH VAD [pre-participation median response 3 (range 2–4), post-participation median 4 (range 3–4), *p* < 0.001]. There were also significant improvements in knowledge assessment before and after the educational module [median number of questions answered pre-participation correctly: 2 (range 0–3); median answered post-participation correctly: 3 (range 2–3); *p* < 0.001]. These gains were maintained at a 6–8-week follow-up. *Conclusions*: High-fidelity simulation with a novel BH VAD simulator, coupled with focused didactics, led to significant improvements in staff confidence and knowledge. This combination of training methods resulted in sustained improvements at 6–8-week follow-up. This approach to staff training can build and solidify knowledge of mechanically supported patients for the nursing staff in a CICU.

## Overview

**Figure 1:**
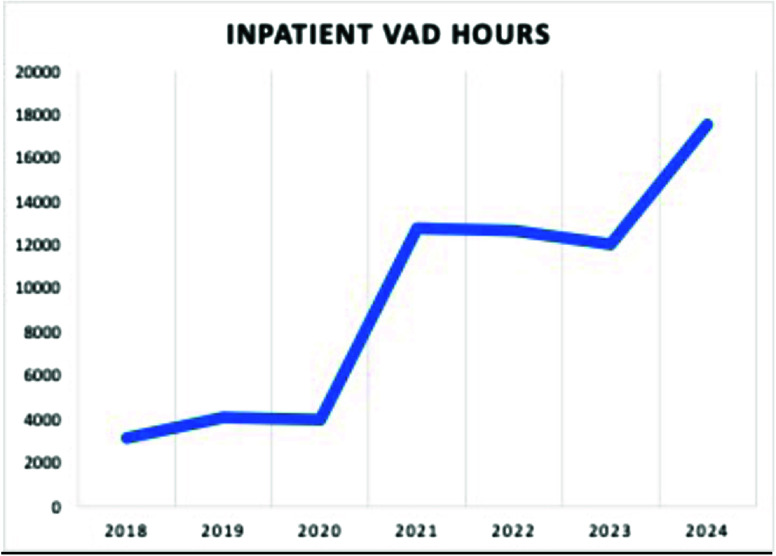
Aggregate annual patient support hours with a ventricular assist device in the cardiothoracic intensive care unit.

Ventricular assist device (VAD) support for pediatric patients has become increasingly common at large centers [1, 2]. Because of this increased prevalence of pediatric VADs, frontline providers in cardiac intensive care units (CICU) must become familiar with clinical assessment of the patient and of the VAD itself. Simulation training has been proven to be an effective method for improving clinical and communication skills in an intensive care setting [3]. With the growth of our institution’s VAD program, the need for additional training for our CICU staff became evident (Figure 1). We designed a nursing-focused educational module, which included a one-hour lecture addressing evaluation of VAD candidacy, patient assessment, and appropriate management of the VAD device, which was presented by our Mechanical Circulatory Support (MCS) coordinators. This was followed by a high-fidelity simulation experience, which allowed the learners to put the content knowledge into action. Assessment of staff confidence and substantive knowledge was performed before and after the sessions, and again 6–8 weeks after the training sessions.

## Materials and methods

### Simulation environment

Simulations took place at a state-of-the-art simulation center in a room designed to simulate a patient room in an intensive care unit. Simulation coordinators can monitor participants via one-way mirrors from a control room, where they can also adjust vital signs on the patient monitors. In-floor conduits allowed for the connection of lines and tubing to the manikin, which remained hidden from the participants.

### Novel Berlin Heart simulator and simulation scenario

**Figure 2:**
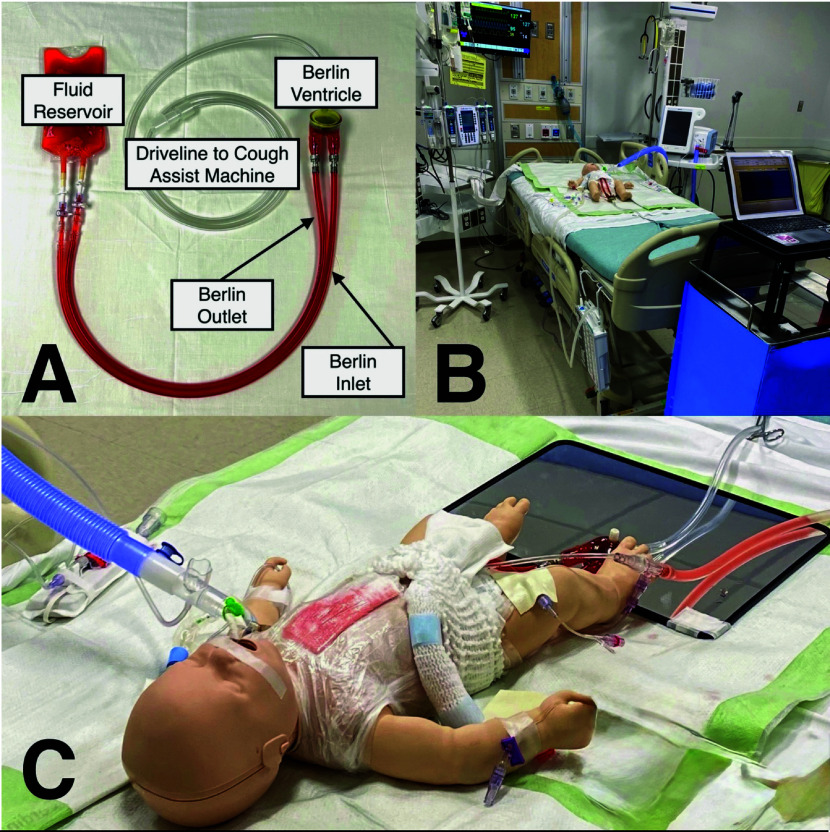
Simulator and Manikin. A) Berlin Heart Simulator with drive line and fluid reservoir. B) Simulated patient environment with all components in place. C) Manikin with mid-sternal dressing saturated with “blood.”

The Berlin Heart ventricular assist device and the IKUS driver (Excor® Pediatric, Berlin Heart Inc, The Woodlands, Texas, USA) are limited resources. At our institution, the Ikus driver is only available for clinical use and not for educational purposes. Additionally, VAD parameters on the IKUS are only adjusted by members of our Mechanical Cardiac Support (MCS) team and not the nursing staff, the use of the IKUS driver and screen was not incorporated in the simulation session. The MCS team received separate training in the use of the IKUS, which was beyond the scope of our nursing education session. To facilitate training in clinical evaluation of the BH VAD, members of the MCS team and Heart Center Simulation team developed a novel BH VAD simulator to mimic the function of the device across different patient physiologic states (Figure 2A). A cough assist machine (CoughAssist T70, Philips Respironics, Pittsburgh, Pennsylvania, USA) was connected to a 10 mL Berlin Heart ventricle to take the place of the IKUS driver delivering positive and negative pressure, moving the silicone membrane at a set rate. The BH VAD ventricle was primed with simulated blood, and inlet and outlet cannula extensions were connected via tubing and one-way stopcocks to a 250 mL reservoir filled with simulated blood. (Figure 2A) Tubing was routed through in-floor conduits from the cough assist machine located in the control room and connected to the drive line of the BH VAD in the simulated patient room (Figure 2B). The cough assist was set to negative 70 cm H_2_O pressure to simulate the fill cycle, positive 70 cmH_2_O pressure to simulate the ejection cycle, and the rate was set to 60 by manipulating the inspiratory and expiratory cycle times. These settings mimic the systolic and diastolic pressure settings of the IKUS. Adjustment of the one-way stopcocks at either the inlet or outlet tubing simulated decreased filling (decreased preload) or ejection (elevated afterload), respectively. These stopcocks were placed in a location out of sight of the participants to prevent forecasting changes to the learners. Care was taken to ensure the 250 mL bag was level with the ventricle during setup to minimize any additional hydrostatic pressure exerted on the silicone membrane. A large mirror was placed under the BH VAD to allow assessment of filling and ejection by all participants in the room.

The simulation scenario was designed to allow for assessment of the BH VAD under baseline physiologic conditions in a non-distressed patient with stable hemodynamics, as well as across 2 different loading conditions – increased afterload and decreased preload. The specific primary learning objectives were to 1) utilize clinical setting, vital signs, and observation to determine inadequate filling or incomplete ejection of the BH VAD, and 2) implement appropriate resuscitation measures. The scenario narrative involved a 14-month-old boy with an anomalous left coronary artery from the pulmonary artery, a type of congenital heart defect often associated with myocardial ischemia, dilation, and dysfunction of the left ventricle. In the scenario, the patient underwent repair, but the left ventricle did not recover, ultimately requiring implantation of a BH VAD.

Phase 1 of the simulation involved the arrival of the patient to the CICU after conversion from a continuous flow VAD to the BH VAD and hand-off from the cardiac anesthesia team. The patient was maintained in a baseline state with stable hemodynamics, and the BH VAD was adjusted to maintain full filling and ejection, allowing the staff initial assessment of the patient and the BH VAD ventricle. Once the team had an opportunity to assess the patient and the BH VAD, the simulation moved to phase 2, where the patient was reported to be agitated. Vital signs showed hypertension and tachycardia; if asked, the simulation coordinator would describe the extremities as cool and clammy. The BH VAD was adjusted to reflect incomplete ejection. Staff were allowed time to confer with the APN and/or fellow. The goal was for them to administer additional sedation and treat remaining hypertension with antihypertensive medications. Once appropriate measures were taken, the simulation was moved to phase 3. During this phase, hidden tubing was used to pump simulated blood into the chest dressing and mediastinal chest tube simulating acute hemorrhage (Figure 2C). Vital signs were adjusted on the monitor to reflect acute hypovolemia with tachycardia and hypotension, and the BH VAD was adjusted to reflect incomplete filling. The goal of this phase was for the team to identify acute hemorrhage and incomplete filling reflecting a decrease in effective circulating volume. The simulation was completed with the administration of fluid and/or simulated blood products.

### Nursing educational module and survey

The CICU conducts quarterly education sessions for the entire nursing staff. These sessions are a combination of didactic content, hands-on skills, and simulations led by the Heart Center Simulation Team. Participants sign up electronically via the institution’s online education tracking system, allowing for easy attendance tracking and documentation of participation. For the BH VAD educational module, each simulation session was limited to a maximum of 6 participants from the nursing team to allow opportunity for hands-on learning and engagement for everyone. Nurse practitioners from the CICU participated in all the simulations as team leaders. Pediatric cardiology or critical care fellows also participated, when possible, as collaborating members of the team.

The didactic portion of the educational module involved a 1-hour lecture on topics related to supporting a patient with the BH VAD, including patient selection, placement, assessment of the ventricle, and anticoagulation. Upon completion of the didactic portion, participants were oriented to the high-fidelity simulation environment, including the manikin, monitors, mechanical ventilator, intravenous (IV) pumps, and available supplies such as a crash cart with medications, and a cooler with simulated blood products (Figure 2B). Roles were assigned to include a charge nurse, bedside nurses, a team leader, and an event recorder, and the simulation progressed as described above. Upon completion of the simulation, 30–45 min were allotted to allow staff to debrief the experience, ask questions regarding the simulation session, and share insights into the care of patients supported with a VAD. Also, during the debriefing, a post-participation survey was administered to assess knowledge acquisition and to obtain impressions from the staff on their experience, including their comfort level caring for a patient supported with a BH VAD and assessing the device for proper function.

Survey questions related to self-reported feeling of confidence in knowledge or practice were scored on a 4-point Likert scale ranging from 1 (disagree) to 4 (strongly agree).

Voluntary and anonymous surveys were administered to all participants prior to the didactic portion of the day (Supplemental Figure 1). The survey was divided into two sections. The first section asked participants to self-report comfort levels on a 4-point Likert scale. The subsequent section asked substantive questions based on video clips and a screenshot of a telemetry screen. The video clips displayed a BH VAD in the fill and eject phases, and participants were asked to determine if the ventricle showed incomplete filling or incomplete ejection. They were asked to identify appropriate interventions based on their assessment of the BH VAD for one of the videos, such as administering a fluid bolus for incomplete filling or administering an antihypertensive medication for incomplete ejection. A voluntary and anonymous post-participation survey was administered at the end of the education day, after the simulation. Participants were asked to rate the same statements as before on a 4-point Likert scale. They were also shown videos of the BH VAD and asked to identify whether the BH VAD had impaired filling or ejection, and what interventions would be appropriate in each situation (Supplemental Figure 2). The post-experience survey concluded with four questions rating the educational experience on a 4-point Likert scale. Voluntary and anonymous follow-up surveys were administered to the nursing staff alone during the 4th quarter education days, 6–8 weeks after the initial experience (Supplemental Figure 3). Knowledge assessment at the time of follow-up had an additional question, precluding statistical comparison with the pre- and post-experience knowledge assessment.

Study data were collected and managed using REDCap electronic data capture tools hosted at The Heart Center, Nationwide Children’s Hospital, Columbus, OH. REDCap (Research Electronic Data Capture) is a secure, web-based software platform designed to support data capture for research studies, providing 1) an intuitive interface for validated data capture; 2) audit trails for tracking data manipulation and export procedures; 3) automated export procedures for seamless data downloads to common statistical packages; and 4) procedures for data integration and interoperability with external sources [4, 5]. Descriptive statistics were used for the demographic data and inpatient VAD hours (Microsoft Excel, Version 16.100.3). Statistical comparisons of the pre- and post-experience surveys were performed using the Mann-Whitney U test for non-parametric data (GraphPad Prism version 10.3.1 for macOS, GraphPad Software, Boston, Massachusetts, USA).

## Results

**Figure 3:**
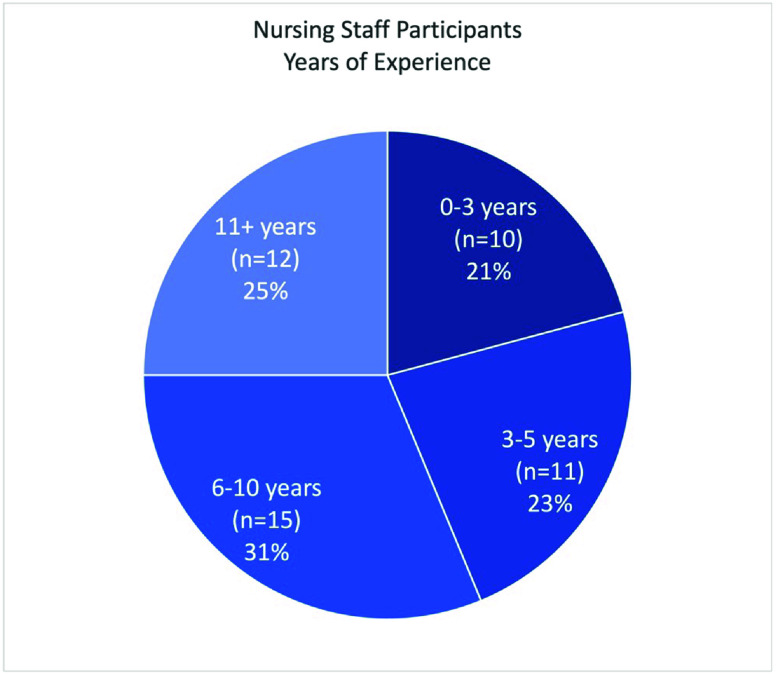
Participant Demographics – Distribution of years of experience among the nursing staff participating in the educational module (number of respondents in parentheses).

**Figure 4:**
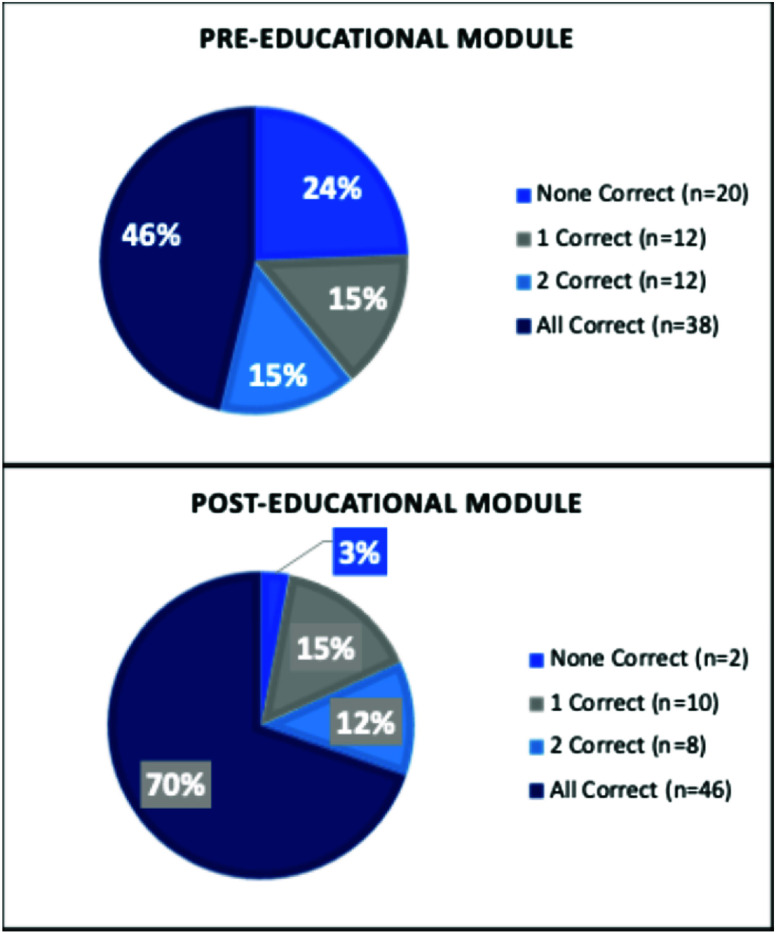
Participant responses to survey questions before and after the educational module and at 6–8-week follow-up (*n* = 82 for Pre, *n* = 66 for Post, *n* = 55 for Follow-up).

**Figure 5:**
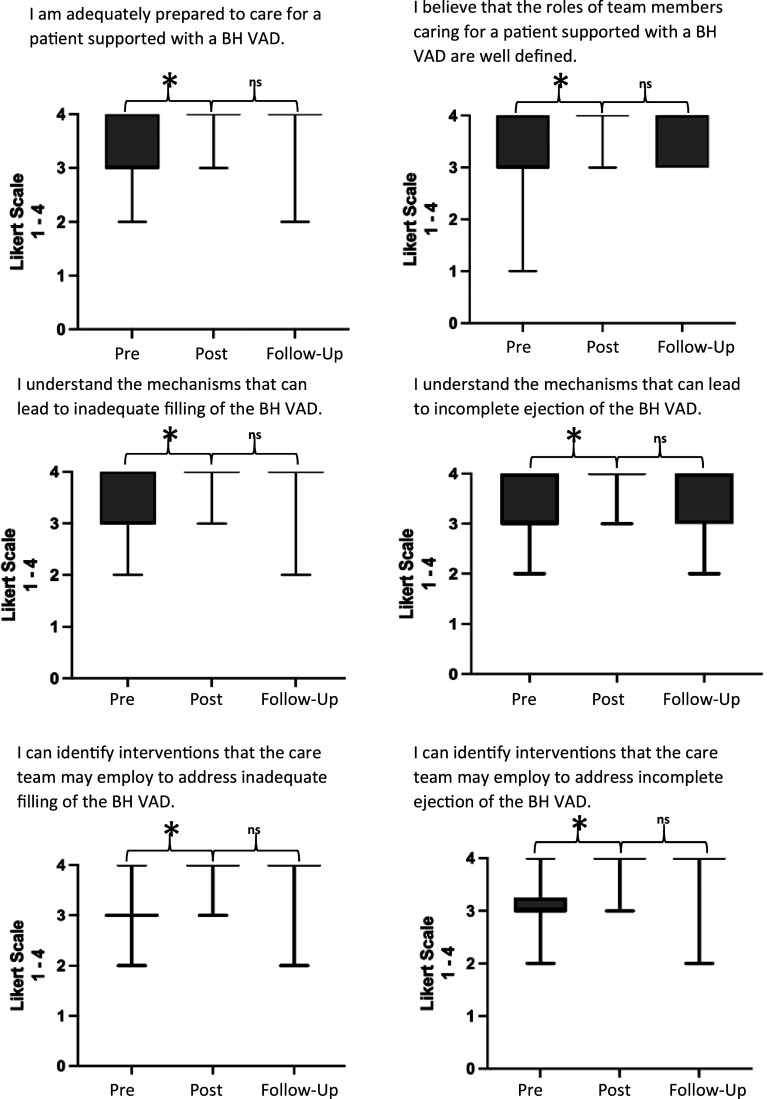
Questions answered correctly on video knowledge assessment before and after didactics and simulation (number of respondents in parentheses).

In the third quarter of 2023, 82 CICU staff members participated in the educational module, including 62 bedside nurses, 13 advanced nurse practitioners (APN), and 7 fellows. Data extracted from the sign-up roster showed that, among the nurse participants, there was an even distribution of experience levels (Figure 3). Given the voluntary nature of the surveys, there was some attrition between the pre-module surveys and the post-module and follow-up surveys. There were 82 respondents to the pre-module survey, 66 respondents to the post-module survey, and 55 respondents to the follow-up survey. Among all participants, there were statistically significant improvements in self-reported feelings of preparedness to care for a patient supported with BH VAD (median pre-participation 3, range 2–4; median post-participation 4, range 3–4; *p* < 0.01), and in comfort assessing the proper functioning of the device (Table 1, Figure 5). Participants’ self-reported understanding of the distinct roles of the team members caring for BH VAD patients also improved significantly (median pre-participation 3, range 1–4; median post-participation 4, range 3–4; *p* < 0.01) (Table 1, Figure 4). Questions directed at participants’ comfort levels assessing the function of the Berlin Heart VAD and identifying interventions to address issues with the VAD also showed significant improvement after the didactic session and simulation (Table 1, Figure 4). The knowledge assessment questions that required participants to watch a video, identify issues with the BH VAD, and identify potential interventions also demonstrated that individuals were able to answer more of the questions correctly after the educational intervention (Table 1; Figure 5). There was no statistically significant difference between post-event and follow-up surveys administered 6–8 weeks after the initial event with respect to self-reported knowledge or feelings of preparedness and understanding (Figure 4).

**Table 1:** Survey results.

	Pre-participation	Post-participation	*p*-value	Follow-up	*p*-value
Survey question[i]	Median (range) (*n* = 82)	Median (range) (*n* = 66)		Median (range) (*n* = 55)	
I am adequately prepared to care for a patient supported with a Berlin Heart VAD.	3 (2–4)	4 (3–4)	<0.01	4 (2–4)	0.43
I believe that the roles of team members caring for a patient supported with a Berlin Heart VAD are well defined.	3 (1–4)	4 (3–4)	<0.01	4 (3–4)	0.08
I am comfortable assessing that a Berlin Heart VAD pump is filling and ejecting adequately.	4 (1–4)	4 (3–4)	<0.01	N/A	N/A
I understand the mechanisms that can lead to inadequate filling of the Berlin Heart VAD.	3 (2–4)	4 (3–4)	<0.01	4 (2–4)	0.9
I can identify interventions that the care team may employ to address inadequate filling of the Berlin Heart VAD.	3 (2–4)	4 (3–4)	<0.01	4 (2–4)	0.6
I understand the mechanisms that can lead to incomplete ejecting of the Berlin Heart VAD.	3 (2–4)	4 (3–4)	<0.01	4 (2–4)	0.08
I can identify interventions that the care team may employ to address incomplete ejecting of the Berlin Heart VAD.	3 (2–4)	4 (3–4)	<0.01	4 (2–4)	0.07
Number of Correct Answers on Knowledge Assessment.[ii]	2 (0–3)	3 (0–3)	<0.01	4 (0–4)	N/A

[i] Likert Scale for Questionnaire: 1 = Disagree, 2 = Somewhat Disagree, 3 = Somewhat Agree, 4 = Strongly Agree.

[ii] 0 = No questions answered correctly; 3 = All questions answered correctly.

## Discussion

While pediatric VADs are less prevalent than adult VADs due to fewer devices, fewer patients, and the unique need to match a pediatric VAD to a wider range of sizes and anatomical variations, their use is on the rise [1, 2]. At our institution, inpatient pediatric VAD hours rose by 320% between 2020 and 2021 (from 3996 h to 12,817 h), with our 2024 numbers remaining over 17,000 h (Figure 1). While our VAD hours have risen exponentially, our unit and nurses can go for extended periods without seeing or caring for a VAD patient. It is reasonable to say that even at 5 years of experience, some nurses may not have taken care of a patient supported by a BH VAD.

As a preparatory step in developing our educational module, we performed a survey of the literature to evaluate prior experience in this area and identify techniques that we could employ in our training modules. We performed two Medline searches for English language articles using the following search terms: “ventricular assist device AND simulation”, and “Berlin Heart AND simulation OR training”. Our query returned 16 articles with the first set of terms and 10 articles with the second. Most of the articles were focused on adult patients and implantable continuous flow devices (Supplemental Table 1). Only 7 articles dealt specifically with the BH VAD, and none of these were focused on staff training or simulation training.

While we were unable to identify any literature on the types of training provided to CICU nursing staff or APNs, Esangbedo *et al*. reported on the results of a survey administered to CICU physicians in 2021 [6]. Among 108 respondents representing 26 CICUs across North America, 86% reported some type of formal training with VADs. Among a variety of modalities identified, it is notable that 77% reported learning at the bedside and only 34% participated in any kind of simulation training. When reporting their comfort levels in managing VADs, 72% felt “comfortable” or “very comfortable”, with 17.6% feeling “neutral”, and 10.2% reporting feeling “uncomfortable” or “very uncomfortable”. While this study addresses a very different population of learners with respect to the assessment and management of VADs, it alludes to the types of training that are employed across several pediatric tertiary care institutions with VAD programs.

Prior training for our nursing staff with the BH VAD relied primarily on didactic sessions and just-in-time bedside training with actual patients. While useful in the moment, the content of this just-in-time training was variable depending on the individual providing training, only captured a small proportion of our nursing staff, and relied primarily on the availability of MCS team members. High-fidelity simulation has proven to be a highly effective means of training and maintaining skills related to low-frequency/high-stakes situations [7]. Since assessment of a BH VAD remains very subjective, experience and repetition in a simulated environment, coupled with didactic discussion and debriefing, allow the staff, and particularly our nurses, to have a greater comfort level when assessing BH VAD mechanics. High-fidelity simulation has been shown to improve assessment skills, teamwork skills, and situational awareness, which are critical when caring for this patient population [3]. Moreover, optimal outcomes for these patients rely on high-functioning, multidisciplinary teams consisting of not only bedside nurses, but also respiratory therapists, nurse practitioners, mechanical circulatory support specialists, and physicians from a variety of specialties. Simulation has been identified as an effective method of fostering situational awareness and communication skills that forge highly effective teams [3, 8, 9]. From a staff perspective, there is a need to match the ongoing training and education for our nursing staff with the growth of our VAD program. The results from this specific educational intervention showed that our staff, predominantly represented by our nurses, not only learned from our content and simulation but also became more comfortable with the assessment of the Berlin Heart VAD and its operation. The use of interprofessional simulation to provide our nursing staff with ongoing education has additional benefits beyond immediate bedside care. Building knowledge and communication skills promotes a sense of autonomy among nurses. Nurses who can practice autonomously within a multidisciplinary team tend to have greater job satisfaction, which may have a positive effect on nursing retention rates, allowing the unit to build a stronger and more knowledgeable team over time [10, 11].

## Limitations

This educational module met its intended goal of increasing staff knowledge and comfort with regard to the clinical assessment and management of the BH VAD. There were some important limitations to our approach, though. Most importantly, to prioritize the voluntary and anonymous nature of the survey, we failed to obtain key demographic data, such as role in the CICU or years of experience, that could have been tied to the responses, allowing a more granular interpretation of the data. Moreover, this approach did not allow us to link surveys, which would have allowed matching data across surveys for each individual and better assess the impact of the educational module. With regards to the substantive questions, without pairing of responses by individual or a more uniform bank of content questions to draw from, adequately assessing retention of the material in the long-term was not possible.

## Conclusion

In summary, high-fidelity simulation with a novel Berlin Heart VAD simulator, coupled with a focused didactic session, results in significant improvements in staff confidence and knowledge when caring for these complex patients. This combination of training methods resulted in significant improvements in knowledge and self-reported confidence in caring for these patients. Our data suggests that there may also be sustained improvements at follow-up 6–8 weeks after the educational session, but the study design precluded a more definitive assessment. This approach to training can build & solidify knowledge of mechanically supported patients for the staff in the CICU.

## Data Availability

Data related to the described project can be obtained by contacting the corresponding author.

## Supplementary material

*Supplemental Figure 1*: Pre-Experience Survey. *Supplemental Figure 2*: Post-Experience Survey. *Supplemental Figure 3*: Follow-up Survey. *Supplemental Table 1*: Medline Search Results. The supplementary material of this article is available at https://ject.edpsciences.org/10.1051/ject/2025058/olm.
