# Supplementary material for: Ventricular assist device simulation to improve staff confidence and knowledge when caring for patients supported with the Berlin heart
Source: J Extra Corpor Technol. 2026 Jun 19;58(2):195–201. doi: 10.1051/ject/2025058 (PMC13281332; doi:10.1051/ject/2025058)
Supplement: Supplementary file 1 — Supplemental Figure 1: Pre-Experience Survey. Supplemental Figure 2: Post-Experience Survey. Supplemental Figure 3: Follow-up Survey. Supplemental Table 1: Medline Search Results. [file ject-58-195-s1.pdf]

Supplemental Figure 1: Pre-Experience Survey

Supplemental Figure 2: Post-Experience Survey

Supplemental Figure 3: Follow-up Survey

Supplemental Table 1: Medline Search Results

Search Terms	Title	Journal	Educational Audience	Age	Device	Context
**Ventricular Assist Device AND Simulation**	Ventricular Assist Device Training and Emergency Management Among Pediatric Cardiac Intensive Care Physicians - Multicenter Cross-Sectional Survey.	World J Pediatr Congenit Heart Surg. 2024 Mar;15(2):202-208	N/A	Pediatric	Various	Identify CICU Attending Physician training with VAD
**Ventricular Assist Device AND Simulation**	Left Ventricular Assist Device Multialarm Emergency: A High-Fidelity Simulation Case for Emergency Medicine Residents.	MedEdPORTAL. 2021 May 5;17:11156	EM Trainees	Adult	HM3	VAD-Specific Emergency Training
**Ventricular Assist Device AND Simulation**	Short-term Retention of Patient and Caregiver Ventricular Assist Device Self-care Skills after Simulation-based Mastery Learning.	Clin Simul Nurs. 2021 Apr;53:1-9	Patient and Caregivers	Adult	HM2/HM3	Educational Research (Patient/Caregiveer Knowledge Retention)
**Ventricular Assist Device AND Simulation**	Human Factors Evaluation of HeartMate 3 Left Ventricular Assist Device Peripherals: An Eye Tracking Supported Simulation Study.	J Med Syst. 2023 May 3;47(1):58	HTX Recipients & Caregivers	Adult	HM3	VAD Development
**Ventricular Assist Device AND Simulation**	Simulation-Based Mastery Learning Improves Patient and Caregiver Ventricular Assist Device Self-Care Skills: A Randomized Pilot Trial.	Circ Cardiovasc Qual Outcomes. 2019 Oct;12(10)	Patient and Caregivers	Adult	HW/HM2/HM3	Educational Research (Comparison of Training Methods)
**Ventricular Assist Device AND Simulation**	Trauma Resuscitation in a Left Ventricular Assist Device Patient: An Emergency Medicine Simulation Scenario.	Cureus. 2017 Oct 13;9(10):e1773	EM Staff	Adult	HW	EM Trauma Team Training
**Ventricular Assist Device AND Simulation**	Improving Left Ventricular Assist Device Competency in Emergency Nurses Using Quarterly Skills Check and Simulation.	J Emerg Nurs. 2025 May;51(3):379-389	EM RN	Adult	HW/HM2/HM3	EM Nursing Competency Training
**Ventricular Assist Device AND Simulation**	Patient, Caregiver, and Clinician Perceptions of Ventricular Assist Device Self-care Education Inform the Development of a Simulation-based Mastery Learning Curriculum.	J Cardiovasc Nurs. 2020 Jan-Feb;35(1):54-65	Patient and Caregivers	Adult	HW/HM	Self/Home Care
**Ventricular Assist Device AND Simulation**	Enhancing Left Ventricular Assist Device Usability: A Comparative Simulation Study of CorWave and HeartMate 3 Peripherals.	ASAIO J. 2025 May 30. doi: 10	HTX Recipients & Lay People	Adult	HM3/CorWave	VAD Development
**Ventricular Assist Device AND Simulation**	Innovative Left Ventricular Assist Device in High-fidelity Patient Simulator.	Cureus. 2020 Apr 21;12(4):e7763	EM Staff	Adult	HM3	Simulator Development
**Ventricular Assist Device AND Simulation**	Mechanical life support algorithm developed by simulation for inpatient emergency management of recipients of implantable left ventricular assist	Resusc Plus. 2022 May 31;10:100254	Inpatient Staff (Stepdown/Ward)	Adult	HM3	Resuscitation Guideline Development

	devices.					
**Ventricular Assist Device AND Simulation**	Transformative Learning, Priming, and Simulation Timing: A Randomized Controlled Pilot Study Among Emergency Medicine Residents.	Cureus. 2023 Oct 24;15(10):e47567	EM Trainees	Adult	HM3	Educational Research (Comparison of Training Methods)
**Ventricular Assist Device AND Simulation**	The Effect of Judge Selection on Standard Setting Using the Mastery Angoff Method during Development of a Ventricular Assist Device Self-Care Curriculum.	Clin Simul Nurs. 2019 Feb;27:39-47.e4	Patient and Caregivers	Adult	HW/HM2	Educational Research (Assessment of Evaluation Methods)
**Ventricular Assist Device AND Simulation**	Paving a Road Home: Developing Education for a Pediatric Home-Going VAD Program.	J Extra Corpor Technol. 2019 Dec;51(4):248-254	Local Care Providers / Patients & Caregivers	Pediatric	HM3	Educational Research (Description of Discharge Training and Local First Responder Training)
**Ventricular Assist Device AND Simulation**	Simulation-Based Education for Urgent Medical Complications Common to the Rehabilitation Setting: An Educational Program for Physical Medicine and Rehabilitation Residents.	PM R. 2019 Dec;11(12):1272-1277	PM&R Trainees	Adult	HM3	Emergency Training (PM&R Trainees, Included in Training for Various Emergencies)
**Ventricular Assist Device AND Simulation**	A Physical Heart Failure Simulation System Utilizing the Total Artificial Heart and Modified Donovan Mock Circulation.	Artif Organs. 2017 Jul;41(7):E52-E65	Future Training and Research	Adult	TAH + MCS Simulator	Simulator Development
**Berlin Heart AND Simulation OR Training**	Successful Implantation of HeartMate3 in a Small Child After Multimodality Imaging Pathway to Assess Feasibility.	ASAIO J. 2024 Jun 1;70(6):e78-e81	N/A	Pediatric	HM3	Case Report (Imaging for Implant Feasibility)
**Berlin Heart AND Simulation OR Training**	Safety issues with an inter-hospital transport of a patient with a Berlin Heart Excor biventircular assist device.	Artif Organs. 2023 Mar;47(3):582-588	N/A	Pediatric	Berlin Heart	Case Report (Transport Safety)
**Berlin Heart AND Simulation OR Training**	Population Pharmacokinetics of Vancomycin in the Pediatric Ventricular Assist Device Population.	Pediatr Crit Care Med. 2020 Aug;21(8):e566-e571	N/A	Pediatric	Various	Pharmacokinetic Study
**Berlin Heart AND Simulation OR Training**	Antithrombin Population Pharmacokinetics in Pediatric Ventricular Assist Device Patients.	Pediatr Crit Care Med. 2019 Dec;20(12):1157-1163	N/A	Pediatric	Various	Pharmacokinetic Study
**Berlin Heart AND Simulation OR Training**	How can LVAD support influence ventricular energetics parameters in advanced heart failure patients? A retrospective study.	Comput Methods Programs Biomed. 2019 Apr;172:117-126	N/A	Pediatric	Berlin Heart	Computer Modeling
**Berlin Heart AND Simulation OR Training**	Computational modelling and evaluation of cardiovascular response under pulsatile impeller pump support.	Interface Focus. 2011 Jun 6;1(3):320-37	N/A	In Silico	Berlin Heart	Computer Modeling

**Berlin Heart AND Simulation OR Training**	Successful Weaning off LVAD Support in an Infant by Pulmonary Artery Banding.	Thorac Cardiovasc Surg Rep. 2025 Jun 10;14(1):e19-e23	N/A	Pediatric	Berlin Heart	Case Report (VAD Management)
**Berlin Heart AND Simulation OR Training**	Cardiac Muscle Training-A New Way of Recognizing and Supporting Recovery for LVAD Patients in the Pediatric Population.	Life (Basel). 2022 Oct 22;12(11):1681	N/A	Pediatric	Berlin Heart	Case Series (VAD Management)
**Berlin Heart AND Simulation OR Training**	Anesthesia for noncardiac procedures for children with a Berlin Heart EXCOR Pediatric Ventricular Assist Device: a case series.	Paediatr Anaesth. 2010 Jul;20(7):647-59	N/A	Pediatric	Berlin Heart	Case Series (Anesthesia Management)
**Berlin Heart AND Simulation OR Training**	Outpatients on biventricular assist devices.	Thorac Cardiovasc Surg. 2001 Oct;49(5):296-9	N/A	Pediatric	Berlin Heart	Case Series (VAD Management)

Abbreviations: CICU – cardiac intensive care unit; EM – emergency medicine; HM2 – HeartMate 2®; HM3 – HeartMate 3®; HTX – heart transplant; HW – HeartWare®; MCS – mechanical cardiac support; PM&R – physical medicine and rehabilitation; RN – registered nurse; TAH – total artificial heart; VAD – ventricular assist device
